# Soy Protein-Based Composite Hydrogels: Physico-Chemical Characterization and In Vitro Cytocompatibility

**DOI:** 10.3390/polym10101159

**Published:** 2018-10-17

**Authors:** Samira Tansaz, Raminder Singh, Iwona Cicha, Aldo R. Boccaccini

**Affiliations:** 1 Institute of Biomaterials, Department of Materials Science and Engineering, University of Erlangen-Nuremberg, 91058 Erlangen, Germany; 2 Section of Experimental Oncology and Nanomedicine, ENT-Department, University Hospital Erlangen University of Erlangen-Nuremberg, 91054 Erlangen, Germany; Raminder.Singh@uk-erlangen.de (R.S.); Iwona.Cicha@uk-erlangen.de (I.C.); 3 Department of Cardiology and Angiology, University Hospital Erlangen, University of Erlangen-Nuremberg, 91054 Erlangen, Germany

**Keywords:** soy protein isolate, composite hydrogels, bioactive glass nanoparticles, biocompatibility, human cells, tissue engineering

## Abstract

Novel composite hydrogels based on the combination of alginate (Alg), soy protein isolate (SPI) and bioactive glass (BG) nanoparticles were developed for soft tissue engineering. Human umbilical vein endothelial cells (HUVEC) and normal human dermal fibroblasts were cultivated on hydrogels for 7, 14 and 21 days. Cell morphology was visualized using fluorescent staining at Days 7 and 14 for fibroblast cells and Days 14 and 21 for HUVEC. Metabolic activity of cells was analyzed using a colorimetric assay (water soluble tetrazolium (WST) assay). Compared to pure Alg, Alg/SPI and Alg/SPI/BG provided superior surfaces for both types of cells, supporting their attachment, growth, spreading and metabolic activity. Fibroblasts showed better colonization and growth on Alg/SPI/BG hydrogels compared to Alg/SPI hydrogels. The results indicate that such novel composite hydrogels might find applications in soft tissue regeneration.

## 1. Introduction

The failure of organs or tissues and the inadequate availability of tissue donors are frequent, difficult and costly issues in human healthcare. Tissue engineering and regenerative medicine are alternative approaches to support, rehabilitate and improve the functionality of damaged tissue by developing engineered biomaterials and biological substitutes [1]. Hydrogels are gaining increasing interest in different biomedical applications such as tissue scaffolds [2], drug delivery [3], cell encapsulation [4] and biofabrication [5] due to their biocompatibility and structural similarity to the extracellular matrix (ECM) [6]. Hydrogels provide an aqueous environment for the cells, having the capability of forwarding nutrients to cells and transporting waste products from cells. Hydrogels may be combined with cell adhesion ligands to promote better cell adhesion and proliferation [7]. Among naturally-derived hydrogel-forming materials, alginate is one of the most popular ones, as it exhibits rapid ionic gelation in moderate pH and temperature conditions, which is suitable for encapsulating cells and different biomolecules such as proteins [8]. However, alginate has a relatively slow degradation profile, and it does not promote efficient cell attachment [9,10,11]. In an attempt to overcome these restrictions of alginate, soy protein isolate (SPI) was considered in this study as a potential biopolymer partner to be combined with alginate. In a previous study, mouse fibroblasts (L929) were cultured on chitosan/SPI films showing better cellular adhesion properties compared to pure chitosan membrane after three days of incubation [12]. The result showed that L929 cells on chitosan films were spherical after three days of incubation; on the other hand, for chitosan/SPI films, due to the incorporation of SPI providing more protein-binding sites, cells were able to attach and proliferate [12]. The alginate/SPI hydrogel films and microcapsules were investigated previously by our group (Tansaz et al. [13]). Cell study results on hydrogel films (with mouse embryotic fibroblast cells) demonstrated the ability of this hybrid hydrogel to promote cell viability and attachment in comparison to pure Alg films [13]. Bioactive glass (BG) particles are being increasingly considered for soft tissue regeneration and wound healing applications [14,15,16]. For instance, Ostomel et al. [17] used silicate glasses as hemostasis-inducing materials for blood clot formation, demonstrating that Ca^+2^ ions releasing from BG can reinforce the coagulation cascade. Also in our previous study [18], the clotting assay on soy protein-BG nanoparticle composite films indicated a higher clotting rate for the composite films compared to the control. Silver-doped mesoporous BG-containing biopolymers induce antibacterial effects and are therefore an alternative choice for healing burn wounds [19]. Moreover, BGs exhibit angiogenic capacity due to the effect of ionic dissolution products’ release [20] and can hence be applied as wound healing materials [15,16]. Jebahi et al. [21] also indicated the positive biological effects of BG containing strontium (Sr) for skin wound repair and regeneration. The results showed that ion release from BG-Sr might have an antioxidant effect. Consequently, a protective action against reactive oxygen species (ROS) was observed in soft tissue surrounding BG-Sr discs and the wounded skin, which resulted in rapid restoration of the structure and functional properties of muscle [21]. In the present study, different silicate nBG particles and also strontium-doped nBG particles were used for the preparation of the composite films. 

The main goal of this study was the fabrication and characterization of a new family of composite hydrogels based on alginate/SPI/BG films for soft tissue engineering. Given the non-animal origin of this polymer blend, less immunogenicity is expected, and alginate/SPI thus represents an alternative biomaterial to animal-derived proteins, which may show immunogenicity and the risk of diseases transmission [22,23,24,25]. Moreover, the effect of incorporating BG nanoparticles into alginate/SPI hydrogel films on their degradation behavior and cytocompatibility with fibroblasts and human umbilical vein endothelial cells (HUVECs) was assessed. 

## 2. Experimental Section

### 2.1. Materials

Sodium alginate (sodium salt of alginic acid from brown algae, suitable for immobilization of micro-organisms, MW 100,000–200,000 g·mol^−1^, guluronic acid content 65–70%) was obtained from Sigma-Aldrich, Munich, Germany. Soy protein isolate was acquired from MP Biomedicals (Eschwege, Germany). Glycerol and calcium chloride di-hydrate (CaCl_2_·2H_2_O) were purchased from VWR international, Belgium. Three different kinds of nanosized bioactive glass (nBG) particles were tested (mean particle size 20–80 nm). The composition of the nBG was very close to (i) 45S5 composition (45 wt % SiO_2_, 24.5 wt % CaO, 24.5 wt % Na_2_O, 6 wt % P_2_O_5_) [26], (ii) 13-93 BG (53 wt % SiO_2_, 20 wt % CaO, 6 wt % Na_2_O, 4 wt % P_2_O_5_, 12 wt % K_2_O, 5 wt % MgO) and (iii) 13-93-5Sr (mean particle size 30–35 nm) composition (53 wt % SiO_2_, 15 wt % CaO, 6 wt % Na_2_O, 4 wt % P_2_O_5_, 12 wt % K_2_O, 5 wt % MgO, 5 wt % SrO). These glass nanopowders were fabricated by the flame spray method [26], and they were characterized in previous studies [27]. 

#### 2.1.1. Sonochemical Preparation of Hydrogels

According to the protocol reported by Silva et al. [28], soy protein isolate (SPI) 2% (*w*/*v*) was dissolved in ultrapure water with glycerol (2 wt %) at 60 °C for approximately 45 min. Meanwhile, 2% (*w*/*v*) sodium alginate (Alg) was dissolved in Dulbecco’s Phosphate-Buffered Saline (PBS), (Gibco, Germany) with a pH range between 7.0 and 7.3 at 37 °C. It should be mentioned that 2% (*w*/*v*) SPI in water at pH around 7 was almost the maximum concentration of SPI that can be dissolved in water, whereas for higher concentration of SPI, the increase or decrease of the pH value of the solution is inevitable. In our study, 2% (*w*/*v*) SPI was used to keep the pH appropriate for the cell culture experiments. Moreover, a lower concentration of glycerol was used here in comparison to the study of Silva et al. [28], due to the previously reported inhibition of cell proliferation by higher concentration of glycerol [29]. After adding different compositions of alginate and soy protein solutions, the ultrasound treatment was performed. This was carried out for three minutes with a pulse duty cycle of “8 s on, 2 s off” according to a previous investigation [28]. The ultrasonic treatment was applied with the probe type ultrasound source “20 kHz Vibracell CV33” from Sonics & Materials, Newtown, CT, USA.

#### 2.1.2. Preparation of Hydrogel Films

The blend solution was transferred into an 8-cm glass Petri dish and left at 37 °C for approximately 30 min to achieve a slight drying of the surface of the hydrogels. This was done to prevent the surface of the hydrogel films from becoming uneven by the addition of CaCl_2_. Afterwards, 0.1 M of calcium chloride solution was poured on the formed hydrogel films and left for 15 min to allow ionic gelation according to the previous protocol [28]. Subsequently, films were washed several times with ultrapure water. Samples that were fitted to the wells of 24-well plates were punched out with a stainless steel cutter with a diameter of 13.5 mm.

#### 2.1.3. Physico-Chemical Characterization of Films

##### 2.1.3.1. Water Uptake

Water uptake is one of the most important properties of hydrogel-based biomaterials. It shows the ability of the hydrogel to absorb and diffuse water or body fluid, which is vital for cells to transfer nutrients and metabolites [30].

For water uptake measurements, the films were previously dried using a critical point dryer (Leica EM CPD300, Leica Microsystems, Wetzlar, Germany). The water uptake ability of the hydrogels was determined by soaking them in Hanks’ balanced salt solution (HBSS, Sigma-Aldrich, Steinheim, Germany). This measurement was carried out up to 3 days at 37 °C, i.e., until the water uptake ability of hydrogel films became steady or started decreasing due to the degradation. The swollen hydrogels were removed at predetermined time points. After washing with ultrapure water and removing the excess water using a filter paper, the hydrogels were weighed with an analytical balance (A&D GX-600, A&D instruments LTD., Tokyo, Japan). The water uptake was calculated according to Equation (1), where *W*_w_ and *W*_d_ are the weights of swollen and dried hydrogels, respectively:

(1) $$\;\mathrm{water}\;\mathrm{uptake}\;\left(\%\right)=\;\frac{Ww-Wd}{Wd}\;\times 100$$

Every experiment was repeated three times (*n* = 3) and, after calculating the water uptake for each sample, the average value was determined.

##### 2.1.3.2. Swelling and Degradation Behavior

The degradation of different composite hydrogels was determined by soaking them in 2 mL HBSS (containing 2% (*v*/*v*) antibiotic to eliminate the risk of bacterial contamination) at 37 °C for a period up to 21 days according to previous studies [28,31,32]. The hydrogels were removed at selected time points (*t* = 1 d, 3 d, 7 d, 14 d, 21 d). The hydrogels were first washed with ultrapure water, and after removing the excess water using a filter paper, the hydrogels were weighed using an analytical balance. The percentage of swelling and degradation was calculated using Equation (2), where *W*_t_ and *W*_i_ are the swollen/degraded weight and the initial weight, respectively:

(2) $$\;\mathrm{weight}\;\mathrm{loss}\;\left(\%\right)=\;\frac{\left(Wt-Wi\right)}{Wi}\;\times 100$$

where positive values are considered as swelling (wt %) and negative values as degradation (wt %). Each experiment was repeated three times (*n* = 3), and after calculating the swelling and degradation for each sample, the average values were determined.

#### 2.1.4. Fourier Transform Infrared Spectroscopy

The chemical structure of the samples was assessed by a Fourier transform infrared (FTIR) spectrometer (Nicolet 6700, Thermo Scientific, Waltham, MA, USA). The analysis was performed under the following conditions: spectral range between 4000 and 530 cm^−1^; the window material was CsI; 32 scans at a resolution of 4 cm^−1^ were recorded. Hydrogel films of Alg and Alg/SPI of different compositions were used to record attenuated total reflectance Fourier transform infrared (ATR FTIR) spectra.

#### 2.1.5. Scanning Electron Microscopy

The morphological analysis of the hydrogel films was performed using scanning electron microscopy (SEM) (Auriga-Zeiss, Jena, Germany). The samples for SEM analysis were dried with a critical point dryer (Leica EM CPD300, Wetzlar, Germany). The samples were sputter coated with gold before SEM examination.

#### 2.1.6. Nano-Indentation

A Piuma nanoindenter instrument (Amsterdam, The Netherlands) was used for the analysis of the effective Young’s modulus of the prepared hydrogels with different Alg(2%)/SPI(2%) ratios (30/70, 50/50, 70/30) on zero day and after 7 and 14 days after immersion in buffer solution. The nano-indentation was carried out with a nano-indentation probe having a cantilever spring constant of 18.200 N/m and a tip radius of 39.00 µm. HBSS was used as medium for the probe calibration. For each hydrogel, several readings were taken at different X, Y positions with a 50–100-µm distance between the points of testing. The results were expressed as the mean value ± standard deviation.

#### 2.1.7. Effect of Incorporation of Bioactive Glass into Alg/SPI Hydrogel Films

The effect of incorporating nBG particles into the Alg/SPI hydrogel on the degradation and cellular response of the films was investigated. Hydrogel films containing nBG were prepared as explained above; however, the addition of 0.5% (*w*/*v*) nBG into the solution was done before performing ultrasound treatment. This concentration of BG was used according to previous studies [28,33]. Moreover, due to the lower viscosity of the blend Alg/SPI solution, this concentration was appropriate to obtain a homogenous solution for the fabrication of hydrogel films. At a higher concentration of nBG, their agglomeration and sedimentation was observed at the bottom of the solution. The effect of nBG particles on water uptake and degradation was assessed. The cytocompatibility of nBG (13-93-5Sr)-containing hydrogels was investigated as described in the next section.

#### 2.1.8. Cell Response to Hydrogels

##### 2.1.8.1. Cells

Primary human fibroblasts (Promocell, Heidelberg, Germany) were cultured in DMEM supplemented with 10% (*v*/*v*) fetal calf serum (FCS) and 1% (*v*/*v*) antibiotic-antimycotic, at 37 °C, with a controlled atmosphere of 5% CO_2_ and 95% relative humidity. A monolayer of fibroblasts in their growth phase (~90% confluence) was detached using trypsin/1 mM ethylenediaminetetraacetic acid (EDTA) (Life Tech., Darmstadt, Germany) in PBS, centrifuged and resuspended in complete cell culture medium. Cells were counted using the trypan blue exclusion method (Sigma-Aldrich) before seeding on hydrogels. Human umbilical vein endothelial cells (HUVECs) were isolated from freshly-collected umbilical cords by a standard technique [34] and grown in ECs growth medium with a growth supplement containing 5% FCS, 4 µL/mL heparin, 10 ng/mL epidermal growth factor, 1 µg/mL hydrocortisone, 50 µg/mL gentamycin sulfate and 50 ng/mL amphotericin B, in a humidified 7.5% CO_2_ atmosphere. The use of human material was approved by the Institutional Ethical Committee on Human Research at the University Hospital Erlangen (Ethical Review Number 85_14 B, from 10 April 2014). All subjects enrolled in this research have given informed consent according to the ethical guidelines. In all experiments, ECs at passage 1–2 were used.

##### 2.1.8.2. Cell Seeding onto 2D Hydrogels

For the cell-compatibility study, circular hydrogel films were prepared using sterilized solution, in a laminar flow hood. To facilitate handling during the cell seeding and assay procedures, the control hydrogels contained 2.5% Alg. Four different sample types were compared, as summarized in Table 1.

The prepared circular hydrogel films were placed in 24-well plates and pre-incubated in DMEM, 10% fetal calf serum (FCS) and 1% antibiotic (penicillin and streptomycin) over night. Afterwards, fibroblasts (75,000 cells/film) or HUVECs (150,000 cells/film) were seeded on top of the hydrogels and incubated in a humidified atmosphere at 95% relative humidity and 7.5% CO_2_ at 37 °C for 7, 14 and 21 days. 

##### 2.1.8.3. Metabolic Activity of Cells

The metabolic activity of both cell types grown on hydrogel films was assessed through the enzymatic conversion of tetrazolium salt (WST-8 assay kit, Sigma Aldrich) after 7, 14 and 21 days of cultivation. Culture medium was completely removed from the samples, and freshly-prepared culture medium was added containing 1 vol% water soluble tetrazolium (WST)-8 solution, followed by incubation for 2 h. Subsequently, 100 mL of supernatant from each sample were transferred into a 96-well plate, and the absorbance was measured at 450 nm in triplicate using a microplate reader.

##### 2.1.8.4. Cell Staining

To assess the morphology of cells after 7, 14 and 21 days of cultivation, cells were washed, fixed and stained with rhodamine phalloidin (Invitrogen, Schwerte, Germany), which selectively stains F-actin, and nuclei were visualized with green nucleic acid stain, SYTOX (Invitrogen, Carlsbad, CA, USA). Fluorescent images were taken with a fluorescence microscope (FM) (Axio Scope A.1, Carl Zeiss Microimaging GmbH, Jena, Germany).

## 3. Results and Discussion

### 3.1. Physico-Chemical Characterization

#### 3.1.1. Water Uptake Behavior

**Figure 1:**
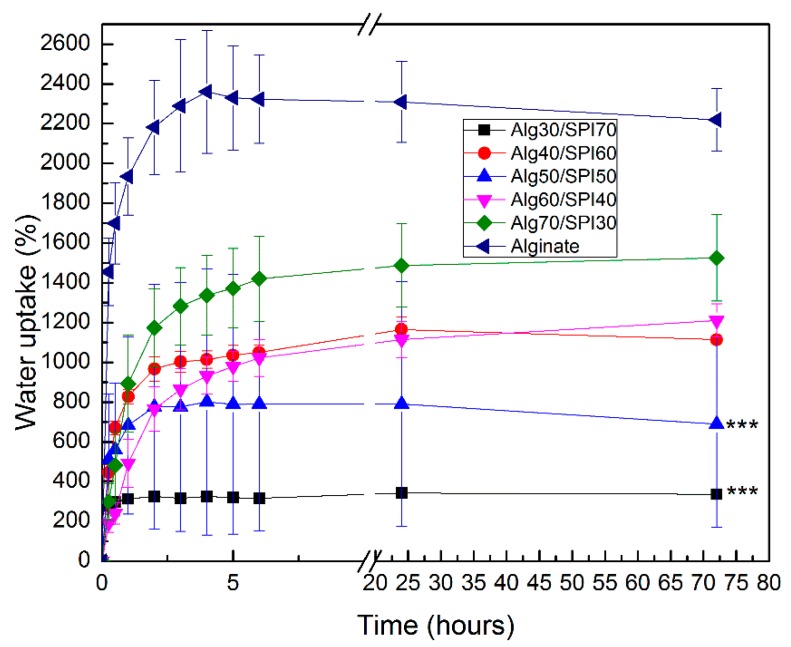
Water uptake ability of different compositions of Alg/SPI hydrogel films in HBSS. Statistically significant differences are indicated in comparison to alginate hydrogel (at 72 h): *** *p* < 0.001 (Bonferroni’s post-hoc test).

The water uptake of hydrogel films of different compositions was analyzed in HBSS. As shown in Figure 1, the water uptake ability for all films increased in the first hours and then became stable. The water uptake was highest for Alg films and decreased with a growing concentration of SPI. This behavior could be due to the higher hydrophilicity of Alg compared to SPI. 

#### 3.1.2. Swelling and Degradation Behavior

**Figure 2:**
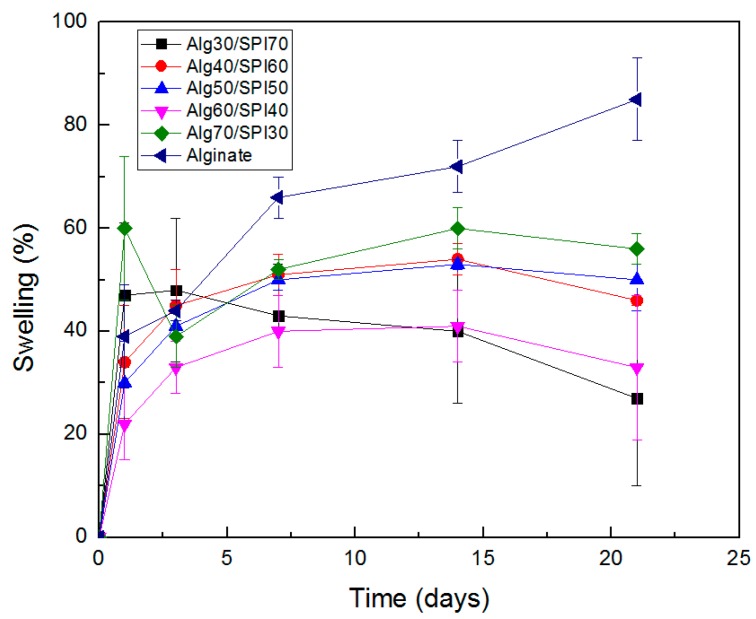
Swelling behavior of different compositions of Alg/SPI hydrogel films.

Figure 2 shows the results of swelling and degradation measurement on different composition of Alg/SPI hydrogel films. At the beginning of incubation, we observed the swelling of all hydrogels, and this ability continued for Alg and Alg/SPI (70/30) until 21 and 14 days, respectively. Alg/SPI (30/70) started to lose weight after three days, and the other compositions started losing weight after 14 days; however, the measured values were still positive, thus showing weight gain. One considerable source of error is the presence of water on the films despite efforts to remove it, and the fact that the produced films are very thin and light, which hinders the accurate measurement in the presence of water. Nevertheless, results clearly showed higher weight loss for Alg/SPI films, especially for the films with a higher concentration of SPI in comparison to the pure alginate films, which is likely due to the release of SPI during the incubation period.

#### 3.1.3. FTIR

**Figure 3:**
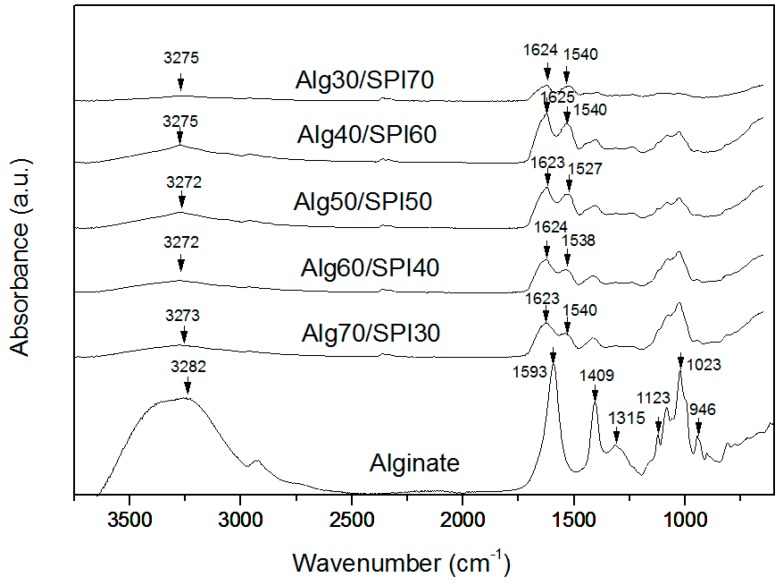
FTIR spectra from different compositions of Alg/SPI hydrogel films. The relevant peaks are discussed in the text.

Figure 3 shows typical FTIR spectra of alginate and all different compositions of Alg/SPI films that were crosslinked by CaCl_2_. The spectrum of the alginate film shows the characteristic absorption bands of the polysaccharide structure. The first relevant peaks are at 1588 cm^−1^ and 1413 cm^−1^, which are assigned to asymmetric and symmetric stretching peaks of carboxylate salt groups of alginate [35]. In addition, the bands around 1316 cm^−1^ (C–O stretching), 1119 cm^−1^ (C–C stretching), 1022 cm^−1^ (C–O–C stretching) and 942 cm^−1^ (C–O stretching) are attributed to the saccharide structure [36,37]. For the Alg/SPI blend spectrum, the typical peaks for SPI are marked in the figure. The SPI spectrum has an absorption band around 3320 cm^−1^, which is related to the stretching vibration of the N–H group (Amide A) [38], and the absorption band at 1670 cm^−1^ is attributed to Amide I (C=O), while Amide II (N–H bending) leads to a peak at 1540 cm^−1^ and Amide III at 1230 cm^−1^ [39,40]. As is observed for Alg/SPI films, the peak of Amide I with small shift is present at 1618 cm^−1^ after the sonication treatment. This effect is likely due to the change of protein conformation to a beta sheet structure, which is usually reported around 1638 cm^−1^ [41]. Silva et al. [28] reported that after sonication treatment, the heating induced by ultrasound could lead to a change in the protein conformation and may allow the soy protein subunits to interact with alginate and form robust networks. 

#### 3.1.4. SEM Analysis

**Figure 4:**
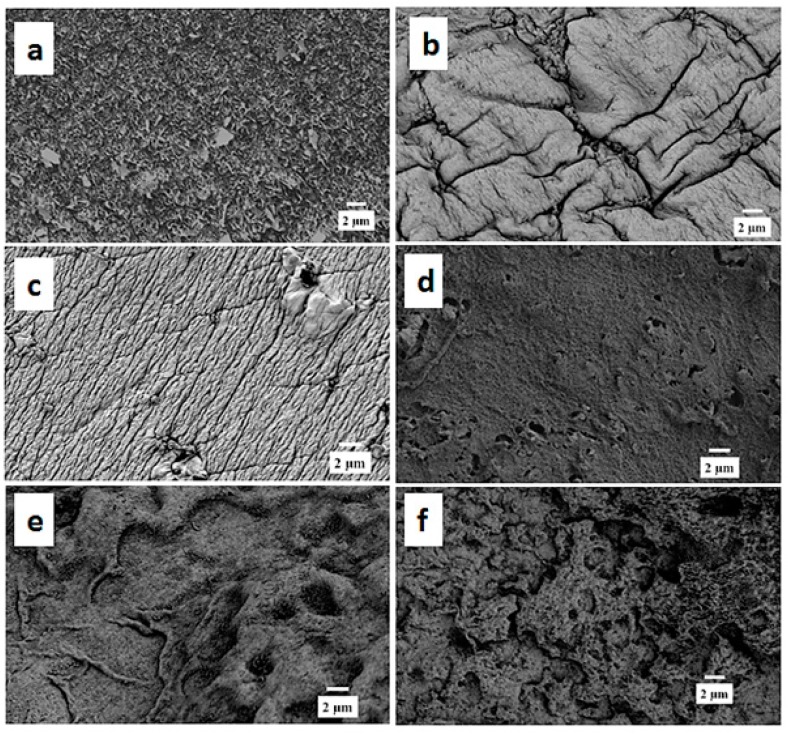
SEM images of different compositions of Alg/SPI hydrogel films: (**a**) Alg, (**b**) 70/30, (**c**) 60/40, (**d**) 50/50, (**e**) 40/60 and (**f**) 30/70 showing different surface morphologies.

As observed in Figure 4, Alg films exhibited the typical morphology with a folded structure in a regular pattern. All different Alg/SPI compositions showed different morphologies compared to Alg. The surface of Alg/SPI (70/30) films had an uneven and rough folded morphology, whereas it was flat and ordered in Alg/SPI (60/40). Alg/SPI (50/50) showed nearly a homogenous porous structure. However, by increasing the concentration of SPI to 60% or 70%, again an uneven and disordered morphology of the film surfaces was observed, which is expected due to the relatively high amount of SPI.

**Figure 5:**
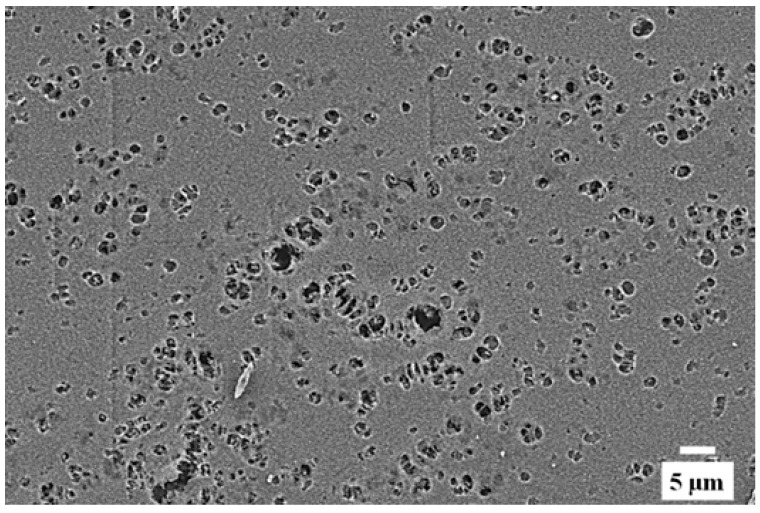
SEM image of Alg/SPI (50/50) hydrogel film after 21 days’ immersion in HBSS showing porosity development.

After 21 days of immersion in HBSS, the surface of Alg/SPI (50/50) hydrogel film was observed by SEM again. As presented in Figure 5, the surface of this hydrogel became smoother, with large voids, which are likely due to the degradation and the release of the soy protein component. These voids are beneficial for cell anchoring and penetration.

#### 3.1.5. Nano Indentation

**Figure 6:**
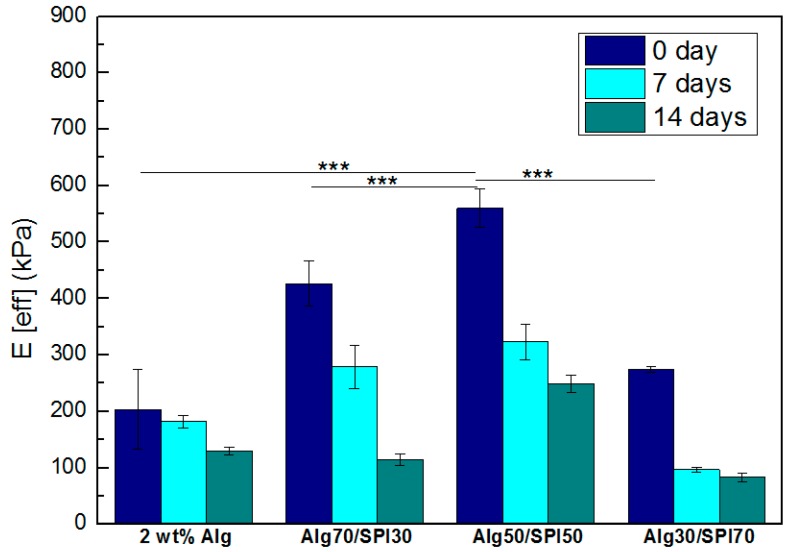
Effective Young’s modulus of prepared hydrogel films with different Alg/SPI ratios on Days zero, 7 and 14. Statistically significant differences are indicated in comparison to Alg/SPI (50/50) hydrogel (day zero): *** *p* < 0.001 (Bonferroni’s post-hoc test).

Nano indentation tests were performed on selected, samples and the effective Young’s modulus of Alg and Alg/SPI hydrogels is plotted in Figure 6. As shown, the incorporation of SPI led to significantly higher stiffness of hydrogel films compared to the pure Alg films. As the amount of SPI increased from 30–50 wt %, there was an increase in Young’s modulus from ~400 kPa–600 kPa. The maximum stiffness was achieved by the Alg/SPI (50/50) film. With a further increase of SPI concentration, stiffness decreased. This is due to the poor miscibility of SPI with Alg, leading to inhomogeneities and to excessive protein content, especially in the case of Alg/SPI (30/70). After seven and 14 days, a reduction of strength can be seen for all different films owing to the hydrogel degradation during time. Degradation was higher for films containing SPI compared to pure Alg films due to the release of SPI.

### 3.2. Effect of Incorporation of Bioactive Glass Nanoparticles into the Alginate/SPI Films

#### 3.2.1. Water Uptake

**Figure 7:**
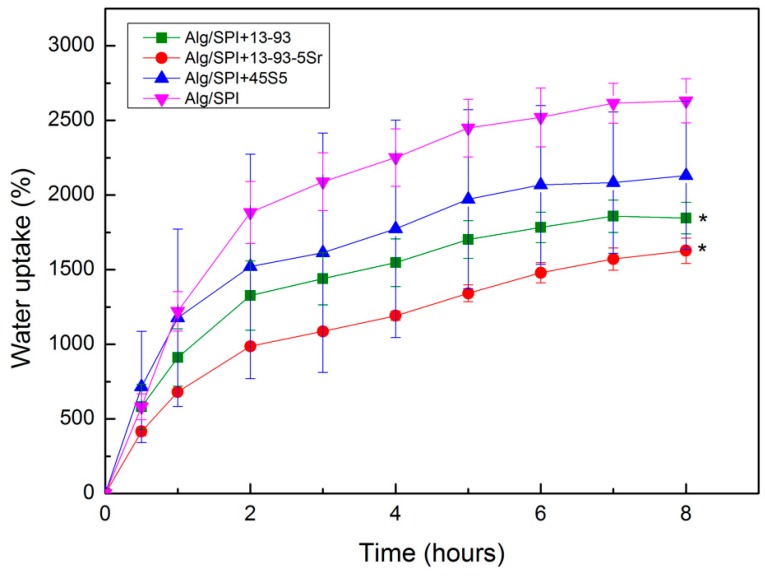
Water uptake of Alg/SPI (50/50) hydrogel films containing different kinds of nBGs (45S5, 13-93 and 13-93-5Sr) up to 8 h. (nBG content = 0.5% (*w*/*v*)). Statistically significant differences are indicated in comparison to Alg/SPI (50/50) hydrogel (after 8 h): * *p* < 0.05 (Bonferroni’s post-hoc test).

Water uptake reflects the ability of the materials to retain and diffuse water, which is an important property of hydrogel-based materials for wound management applications [42]. Figure 7 shows the water uptake behavior of the films either without nBGs or with different kinds of nBGs, over time. Generally, a strong gain of weight can be observed, with an increase of up to 2600% after 8 h for the Alg/SPI (50/50) films. However, the addition of nBG led to a reduction in water uptake capacity compared to the control (pure Alg/SPI (50/50) films). These results are in agreement with the findings of Silva et al. [28]. The observed differences in water uptake ability may be due to strong interactions between Alg/SPI with nBG. Moreover, a decrease in pore size of hydrogel films due to the addition of nBG could lead to reduced water uptake. The addition of nBG in the hydrogel films might fill up the hydrogel porosity, thus providing less space for absorbing water.

#### 3.2.2. Swelling and Degradation Behavior

**Figure 8:**
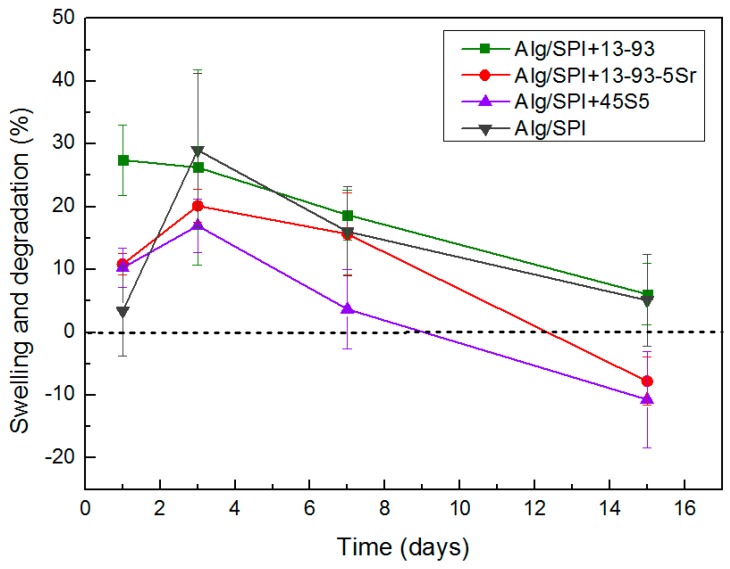
Swelling and degradation behavior of Alg/SPI (50/50) hydrogel films containing different kinds of BGs (45S5, 13-93 and 13-93-5Sr) up to 15 days.

The swelling and degradation behavior of samples were studied over a period of 15 days. Degradation was determined according to Equation (2); positive values indicate swelling, and negative value are considered as degradation. As shown in Figure 8, there was an average weight gain in all samples until Day 3 (positive weight value), followed by a degradation effect observed from Day 7, which increased at Day 15. After two weeks of incubation in HBSS, the degradation of all films was similar, which might be due to the measurement error owing to the presence of water on the very thin and light films despite efforts to remove it. Glass 13-93 has a comparatively higher SiO_2_ content compared to 45S5 BG, and also due to the additional network modifiers, such as K_2_O and MgO, 13-93 BG is less reactive than 45S5 BG, which leads to a reduced degradation behavior of films containing 13-93 BG. Moreover, due to the release of strontium ions from hydrogel films containing 13-93-5Sr BG into the medium, a higher degradation was observed in these samples as compared with the hydrogel films containing 13-93 BG. Sr-containing 13-93 BG was previously shown to degrade more rapidly than 13-93 BG in cell culture medium [27].

**Figure 9:**
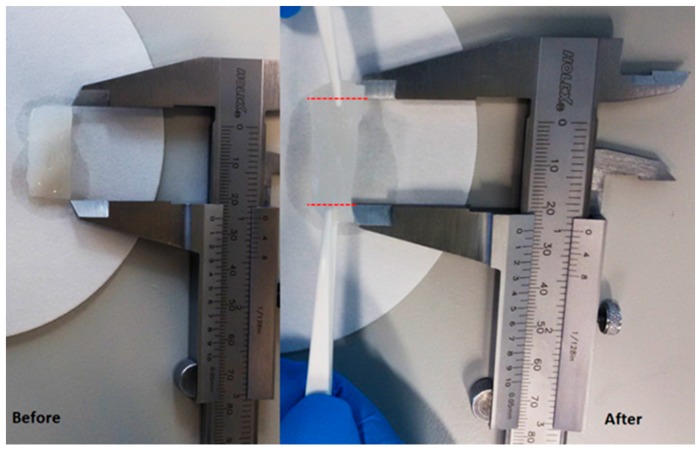
Alg/SPI (50/50) hydrogel film containing nBG (13-93-5Sr) before and after applying a stretching force manually using tweezers. Red dashed lines show the qualitative elongation of the hydrogel film.

To assess (qualitatively) the mechanical stability of Alg/SPI (50/50) hydrogels containing nBG, a stretching force was applied manually using tweezers, as shown in Figure 9. The film had an acceptable mechanical strength and deformation capability, which indicates suitability to be used as wound dressing material. 

### 3.3. Cell-Material Interactions Using Fibroblasts and HUVECs

**Figure 10:**
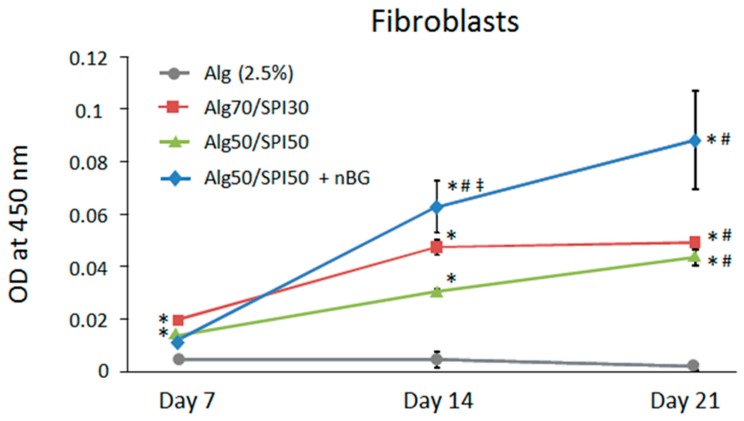
Metabolic activity of fibroblasts on Alg (2.5%), Alg/SPI (70/30), Alg/SPI (50/50) and Alg/SPI (50/50) + nBG hydrogel films after 7, 14 and 21 days of cultivation. * *p* < 0.05 versus pure Alg on the respective day; # *p* < 0.05 versus the same type of hydrogel on Day 7; ‡ *p* < 0.05 versus Alg50/SPI50 without nBG on Day 14. One-way ANOVA on ranks with pairwise comparison using the Student–Newman–Keuls test.

To test the in vitro biocompatibility of the developed SPI-based hydrogel films, cell–material interactions were investigated using primary human cells, fibroblasts and HUVECs. Gorustovich et al. [20] have indicated that BG can stimulate fibroblast cells to secret angiogenic growth factors, and dissolution products from BG can promote endothelial cell proliferation [43,44,45,46,47]. To investigate the metabolism of the cells on hydrogel films containing Alg, Alg/SPI and Alg/SPI containing nBG after 7, 14 and 21 days of incubation, the WST-8 assay was performed. As presented in Figure 10, the metabolic activity of fibroblasts grown on Alg/SPI hydrogel films at both concentrations of 50/50 and 70/30 was significantly higher compared to that of cells grown on pure Alg over 21 days of culture. This effect was not unexpected, as Alg does not support cell adhesion, and the presence of protein isolate in Alg/SPI hydrogels provides binding motifs for cells. Interestingly, in fibroblasts grown on Alg/SPI films containing nBG, the metabolic activity was further increased at later time points (Day 14 and Day 21), both in comparison to pure Alg and to Alg/SPI without nBG. 

**Figure 11:**
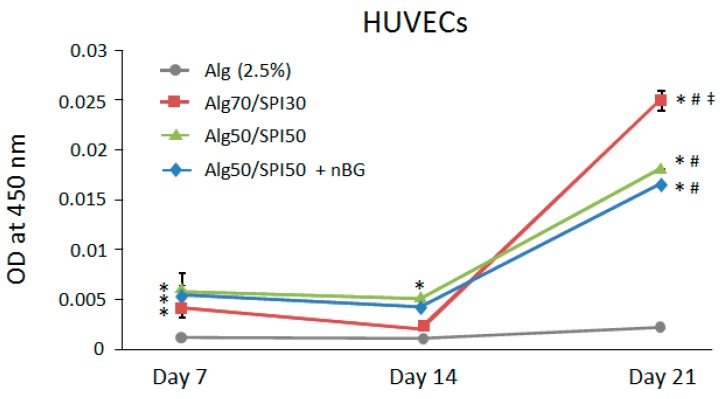
Metabolic activity of HUVECs on Alg (2.5%), Alg/SPI (70/30), Alg/SPI (50/50) and Alg/SPI (50/50) + nBG hydrogel films after 7, 14 and 21 days of cultivation. * *p* < 0.05 versus pure Alg on the respective day; # *p* < 0.05 versus the same type of hydrogel on Day 14; ‡ *p* < 0.05 versus Alg50/SPI50 and Alg50/SPI50 + nBG on Day 21. One-way ANOVA on ranks with pairwise comparison using Student–Newman–Keuls test.

Also in case of HUVECs, as shown in Figure 11, Alg/SPI hydrogel films with and without BG supported metabolic activity of cells in comparison to pure Alg films, which was likely due to the presence of cell binding peptide in the Alg/SPI films. Notably, the presence on nBG in the Alg/SPI hydrogels did not further increase metabolic activity of HUVECs. Compared to fibroblasts, HUVECs showed the largest increase in metabolic activity after 21 days of cultivation. The reason for this result could be that the gradual degradation of the hydrogel during incubation causes an increased exposure of SPI, which, in parallel with the altered surface topography of the hydrogel, enables better cell proliferation and activity.

**Figure 12:**
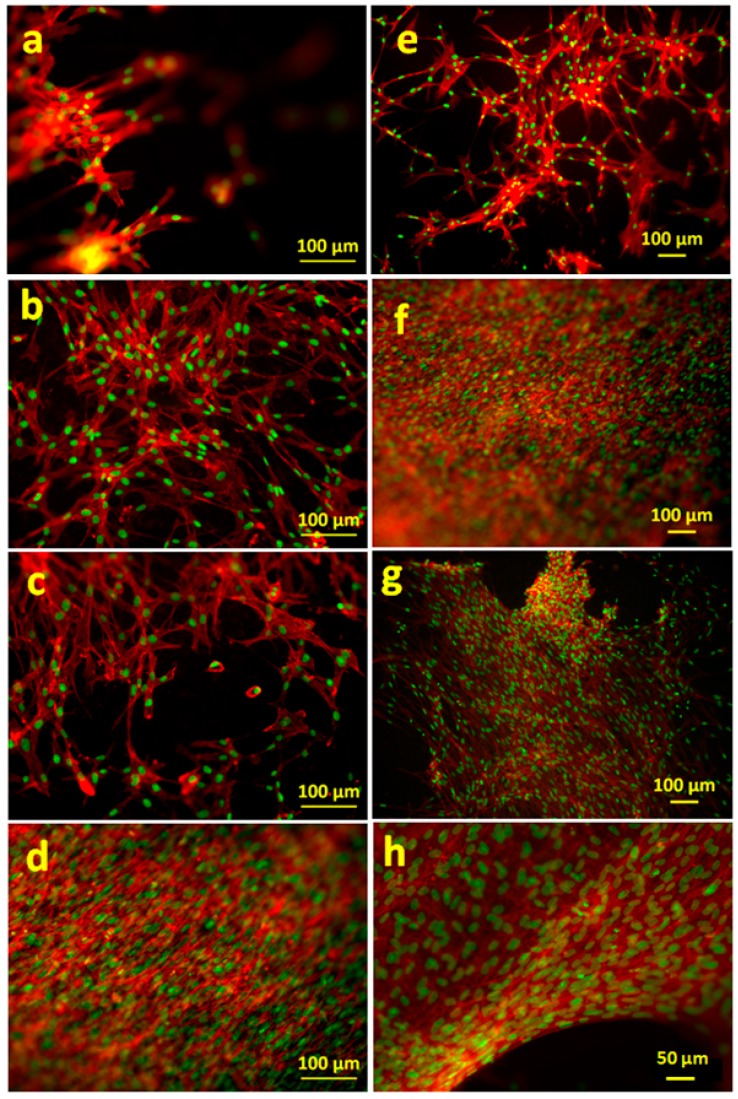
FM images of fibroblasts adhered on (**a**,**e**) Alg (2.5%), (**b**,**f**) Alg/SPI (~70/30), (**c**,**g**) Alg/SPI (50/50) and (**d**,**h**) Alg/SPI (50/50) + 0.5%(*w*/*v*) BG after seven days (left column) and 14 days (right column) of cultivation. The cells were stained for F-actin (red) and nuclei (green).

**Figure 13:**
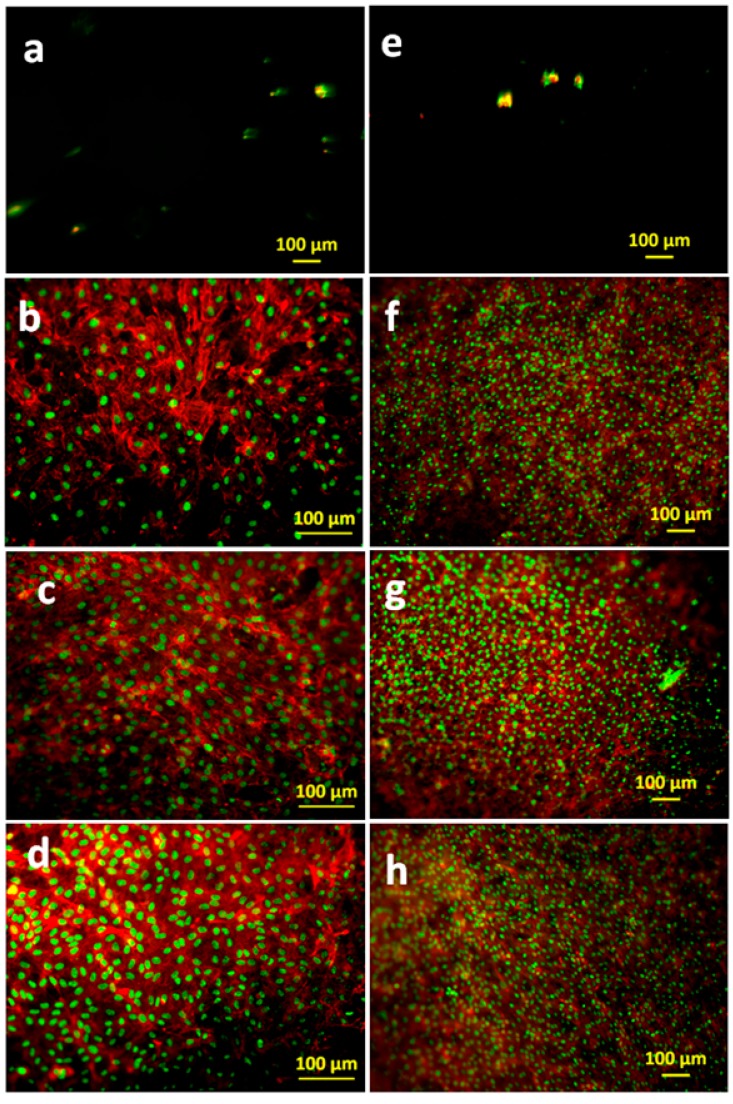
FM images of HUVECs adhered on (**a**,**e**) Alg (2.5%), (**b**,**f**) Alg/SPI (~70/30), (**c**,**g**) Alg/SPI (50/50) and (**d**,**h**) Alg/SPI (50/50) + 0.5%(*w*/*v*) BG after 14 days (left column) and 21 days (right column) of cultivation. The cells were stained for F-actin (red) and nuclei (green).

The cell morphology was visualized by actin cytoskeleton staining, using rhodamine phalloidin, as shown in Figure 12 and Figure 13. As expected from the metabolic activity results, poor anchorage and clustering of fibroblasts were observed on pure alginate hydrogels due to the reduced cell-material interaction. This effect was more pronounced in HUVECs, where very low numbers of cells were detected on Alg hydrogel, as compared to Alg/SPI (both concentrations) and to Alg/SPI + nBG films.

Over seven days of cultivation, the most desirable conditions for fibroblast growth and spreading were observed on Alg/SPI + nBG. Fibroblasts showed (Figure 12) the best surface coverage and spindle-like morphology on these hydrogels and formed multilayers similar to fibroblasts grown on standard cell culture plastic. Correspondingly, their metabolic activity was significantly higher than on pure Alg and Alg/SPI (both concentrations). This effect became even more evident after 14 days. Although no SEM images were taken after the addition of nBG, we observed that upon the addition of SPI to Alg films, soy protein filled the porosity of alginate structures. As can be seen in Figure 4, a smoother surface was achieved for the Alg/SPI (50/50). Because nBG can fill in the pores, the addition of nBG is expected to enhance this effect, which may promote the initial attachment of fibroblasts [48,49]. Interestingly, although after seven days of cultivation, there were fewer cells observed on the surface of both concentrations of Alg/SPI hydrogels in comparison to the Alg/SPI + nBG, after 14 days of cultivation, fibroblasts covered the surface of Alg/SPI hydrogels in a similar way to Alg/SPI + nBG. Cultivated cells proliferated and covered the whole surface of the hydrogel films. This behavior can be ascribed to the ion release from nBG inside the hydrogel films over the incubation time, which may promote cell proliferation [46]. Furthermore, Day et al. [47] demonstrated higher cell proliferation for poly(d,l-lactide-*co*-glycolide) composite foams containing BG compared to the foams without BG. Moreover, due to the progressive release of SPI and BG dissolution over the cultivation period, the porosity and surface topography gradually increased, reaching micrometer range, which can facilitate cell growth. 

Similar results were observed for HUVECs. After 14 days, the surface of the Alg/SPI (50/50) hydrogel films was covered completely with HUVECs. Moreover, addition of nBG showed no toxicity for this type of cells, as also demonstrated by the results of the viability assay. In addition to the higher cell viability on Alg/SPI films, the density of HUVECs that covered the surface of the composite hydrogels was higher than that on alginate films. This suggests the superior cell-material interaction, which is likely due to the chemical bonding between peptides of SPI biomolecules and cells. The same results have been observed for hybrid Alg/protein hydrogel films such as gelatin, silk fibroin and keratin compared to pure Alg hydrogel films [31,32,50]. In comparison to pure Alg hydrogel films, Alg/keratin, Alg/gelatin and Alg/silk fibroin hydrogel films provided superb surfaces for HUVEC, supporting their attachment and proliferation.

Silva et al. have combined alginate with keratin [31], silk fibroin [32] and elastin [51] for tissue engineering applications. Compared with the hybrid hydrogel based on alginate/keratin, alginate/silk fibroin and alginate/elastin, the degradation rate for both SPI containing hydrogel films in this study was found to be lower than that of alginate films over 21 days (less than 40%). However, in the present study, the weight loss of Alg/SPI (50/50) films was higher than that of alginate films, and it was around 60% and 90% over 21 days in HBSS and DMEM, respectively. This result indicates that due to the presence of soy protein in the films, the drawback of an unfavorable slow degradation kinetic of alginate can be overcome by the release of soy protein and providing voids, as shown in Figure 5. The release kinetics of soy protein from the hybrid hydrogel films over the incubation time in HBSS was also described by Silva et al. [28]. It must be noted that this process was shown to occur gradually, whereby about 25% of protein is released within seven days and about 37% within 21 days of incubation. Moreover, cytocompatibility studies of both HUVECs and fibroblasts cultured on the Alg/SPI hydrogel films indicated higher cell-compatibility compared to pure Alg, similar to the result of other hybrid Alg/proteins (silk fibroin, keratin, gelatin and elastin) hydrogel films [50]. Therefore, according to the low immunogenicity and long storage capability of SPI and also its being inexpensive and abundant, this plant-derived protein represents an attractive alternative to overcome the limitations of Alg for tissue engineering applications such as inappropriate stiffness, lack of cellular adhesion motifs and very slow and uncontrolled degradation kinetics.

## 4. Conclusions

Hydrogel films based on the combination of alginate and soy protein isolate were successfully prepared. The results illustrated that Alg/SPI biopolymer had higher degradability than pure Alg owing to the SPI release of the soy protein component. Nano indentation confirmed that the incorporation of SPI to the Alg hydrogel increases the effective Young’s modulus. Furthermore, cell studies on hydrogel films demonstrated the ability of these hybrid hydrogels to promote cell viability, attachment and proliferation in comparison to pure Alg films, which can be ascribed to cell adhesive molecules present in soy protein. The addition of BG to the hydrogel films enhanced cell viability and cell proliferation for fibroblasts and HUVECs compared to Alg/SPI films. The results of the present study show that the combination of alginate with soy protein isolate leads to better degradability of pure alginate. Moreover, improved fibroblast attachment compared to pure alginate was further positively affected by the presence of nBG. Hence, this combination of biopolymers and nBG represents an attractive system for wound dressing applications and soft tissue engineering.

## Figures and Tables

**Table 1:** Composition of hydrogel films used in the cell compatibility studies. Alg, alginate; SPI, soy protein isolate; nBG, nanosized bioactive glass.

Formulation	Compound 1	Compound 2	Compound 3	Final Composition
Alg	Alg 2.5%	-	-	100 (2.5%)
Alg/SPI (70/30)	Alg 5%	SPI 2%	-	50/50 (2.5%/1%)
Alg/SPI (50/50)	Alg 2%	SPI 2%	-	50/50 (1%/1%)
Alg/SPI (50/50) + nBG	Alg 2%	SPI 2%	nBG	50/50 (1%/1%) + 0.5% (*w*/*v*)
